# Efficacy of Dual N-Back Working Memory Training in Children and Adolescents with Autism Spectrum Disorder: A Randomized Controlled Trial

**DOI:** 10.1192/j.eurpsy.2026.10570

**Published:** 2026-07-31

**Authors:** N. M. Kong

**Affiliations:** Psychiatry, Queen Mary Hospital, Hong Kong, Hong Kong

## Abstract

**Introduction:**

Working memory (WM) deficits are a core cognitive feature of Autism Spectrum Disorder (ASD), contributing to significant functional impairments. Computerized cognitive training, such as the dual n-back task, represents a promising intervention, but evidence from rigorous randomized controlled trials (RCTs) in ASD is scarce.

**Objectives:**

To investigate the efficacy and durability of a dual n-back training program on WM function in children and adolescents with ASD.

**Methods:**

In this registered RCT, 69 participants with ASD (aged 10-15) were randomly assigned to either an intervention group (n=35), which completed adaptive dual 2-back training, or an active control group (n=34), which completed a 0-back task. Training was conducted daily for two weeks. Outcomes were assessed at baseline, post-intervention, and at a 3-month follow-up using performance-based WM measures (Dual N-Back Task, Digit Span) and parent-reported executive functioning (Behaviour Rating Inventory of Executive Function, BRIEF).

**Results:**

Intent-to-treat analysis revealed significant and sustained improvements in the intervention group compared to the active control. The intervention group showed significantly greater gains in both verbal and visuospatial WM accuracy on the dual n-back task (all p < 0.05). These near-transfer effects were maintained at the 3-month follow-up. Furthermore, parents of children in the intervention group reported significant improvements in everyday working memory and emotional regulation on the BRIEF (p < 0.001), with large effect sizes. In contrast, minimal changes were observed on broader behavioural symptom measures.

**Conclusions:**

Dual n-back training is an effective and durable intervention for enhancing working memory capacity and everyday executive functioning in children and adolescents with ASD. This training protocol shows promise as a targeted cognitive intervention within a comprehensive treatment plan.

**Disclosure of Interest:**

None Declared

